# Screening Beyond Dependence: At-Risk Drinking and Psychosocial Correlates in the Heart Transplant Population

**DOI:** 10.3390/diagnostics15212812

**Published:** 2025-11-06

**Authors:** Alexandra Assabiny, Zsófia Ocsovszky, Blanka Ehrenberger, Orsolya Papp-Zipernovszky, József Otohal, Kamilla Marjai, József Rácz, Béla Merkely, Beáta Dávid

**Affiliations:** 1 Heart and Vascular Centre, Semmelweis University, 1085 Budapest, Hungary; ocsovszky.zsofia@semmelweis.hu (Z.O.); ehrenberger.blanka@semmelweis.hu (B.E.); papp.zipernovszky.orsolya@semmelweis.hu (O.P.-Z.); otohal.jozsef@semmelweis.hu (J.O.); rector@semmelweis.hu (B.M.); 2 Institute of Psychology, Eötvös Loránd University, 1053 Budapest, Hungary; racz.jozsef@semmelweis.hu; 3 Department of Addictology, Faculty of Health Sciences, Semmelweis University, 1085 Budapest, Hungary; marjai.kamilla@semmelweis.hu; 4 Department of Surgery, Transplantation and Gastroenterology, Semmelweis University, 1085 Budapest, Hungary; 5 Institute of Mental Health, Semmelweis University, 1085 Budapest, Hungary; david.bea@semmelweis.hu

**Keywords:** heart transplantation, alcohol consumption, at-risk drinking, adherence

## Abstract

**Background/Objectives**: Psychosocial factors (e.g., adherence, substance use) contribute to increased morbidity and mortality after heart transplantation. We investigated alcohol consumption patterns and their associations with psychosocial factors in adults, who underwent heart transplantation surgery (HTX recipients). **Methods**: Our cross-sectional study was conducted at the Semmelweis University Heart and Vascular Centre between 2023 and 2025. In total, 201 HTX recipients (75.6% male, mean age: 56.33 ± 11.46 years) completed the Alcohol Use Disorders Identification Test (AUDIT), Brief Health Literacy Screening Tool (BRIEF), Medication Adherence Report Scale (MARS-5) modified to immunosuppressive medication, and 9-item Beck Depression Inventory (BDI-9). Statistical analysis included Pearson’s correlation tests and Multivariate Regression Analyses. **Results**: The AUDIT had a higher proportion of non-evaluable responses than other questionnaires (AUDIT 19.9% vs. 5.5–9%), with 41.0% of the participants abstinent, 54.7% low-risk, 4.3% medium-risk, and 6.5% at-risk drinkers. AUDIT correlated negatively with MARS-5 (r = −0.326; *p* = 0.000) and positively with BDI-9 (r = 0.208; *p* = 0.010). At-risk drinking was associated with a lower MARS-5 (r = −0.231; *p* = 0.002). Multivariate regression models significantly predicted the AUDIT (F = 5.106; *p* < 0.001, R^2^ = 0.216) and AUDIT-C (F = 3.804; *p* = 0.002; R^2^ = 0.146), with sex and adherence as independent predictors. **Conclusions**: The high proportion of non-evaluable AUDIT responses suggests limitations in multi-questionnaire use but does not diminish its clinical relevance. The presence of 6.5% at-risk and 4.3% medium-risk drinkers highlights the relevance of consumption pattern screening, beyond diagnosing alcohol use disorder. Associations between AUDIT, MARS-5, and BDI-9 emphasize the necessity for multidisciplinary care.

## 1. Introduction

Alcohol use disorder and heart failure are major public health issues, and the relationship between them manifests in multiple contexts. Alcohol is an etiological factor of cardiomyopathy and contributes to hypertension and atrial arrhythmias. Furthermore, binge drinking is an independent risk factor for atrial fibrillation [1,2,3,4]. Beyond the diagnosis of alcohol use disorder, the evidence of the effects of consumption patterns has been rapidly increasing in the last 10 years. Although some studies have suggested that low levels of alcohol consumption may confer a modest protective effect against cardiovascular disease risk, several reviews found that this protective effect is negated in the presence of episodes of heavy alcohol consumption [5]. Newer evidence has also emerged, showing that the health risk becomes apparent at lower levels of alcohol intake [6]; therefore, the current statement of the World Health Organization claims that no level of alcohol consumption can be considered safe for health [5].

The gold standard therapy for advanced heart failure is heart transplantation, where the recipients represent the most complex subset of the cardiology patient population. Most of these individuals require the full spectrum of cardiological interventions throughout their disease trajectory, including the stepwise management of progressive heart failure and a rigorous evaluation process for transplant eligibility. In the post-transplant period, lifelong adherence to both immunosuppressive pharmacological and nonpharmacological therapy is crucial. Nonadherence has been linked to an increased risk of post-transplant coronary artery disease, late acute rejection, and higher mortality rates [7]. Beyond somatic status, transplantation-related outcomes such as recovery and survival can be significantly predicted by pretransplant psychosocial characteristics [7,8]. Pretransplant substance use can increase the candidate’s risk for poor post-transplant outcomes: “excessive” alcohol use increases the risk of post-transplant mortality, poor medication adherence, and maladaptive outcomes of the transplantation [7]. Pretransplant depression also increases the risk of post-transplant nonadherence and mortality [7]. However, as a protective factor, sufficient caregiver and social support systems can improve medication adherence, emotional adjustment, and overall outcome, including mental health problems [7].

The guidelines of the International Society for Heart and Lung Transplantation recommend [7] a psychosocial evaluation process before the surgery. Alcohol consumption screening tools are suitable for use in transplant patients. Comparative analyses suggest that the Alcohol Use Disorder Identification Test (AUDIT) is the most reliable, as it effectively identifies both hazardous drinking and alcohol dependence [9]. Therefore, its application should be considered in the evaluation process of transplant candidates [8]. Excessive alcohol use is an absolute contraindication of transplantation, but the guidelines do not provide a definition of excessive use, instead citing national guidelines [7,8].

The Diagnostic and Statistical Manual of Mental Disorders—Text Revision [10] defines alcohol use disorder (AUD), as well as its specific code in electronic health records; consequently, more data relating to AUD are available than data on consumption patterns in the heart transplant population. An Italian study examining 83 heart transplant candidates found that 9.6% had a history of alcohol abuse, although no patients had a diagnosis at the time of their evaluation [11].

Research data relating to alcohol consumption after solid organ transplantation is mostly available in the liver transplant population, and recommendations in heart transplantation guidelines are mainly based on this [7,12,13]. Nevertheless, correlates of alcohol use or at-risk drinking effects are underestimated in the heart transplant population [14].

Our aim was to assess the alcohol consumption patterns of heart transplant recipients, who were cared for at the Semmelweis University Heart and Vascular Center, the prevalence of their at-risk drinking, and the correlations between alcohol consumption and relevant psychosocial factors, such as depression, medication adherence, and health literacy.

## 2. Materials and Methods

### 2.1. Participants

Our single-center cross-sectional study was approved by the local ethics committees (Semmelweis University Regional and Institutional Committee of Science and Research Ethics Nr. SE RKEB 166/2023, 8 August 2023). We enrolled adult heart transplant patients over 18 years of age who were regularly cared for by the Heart Transplantation Working Group of Semmelweis University Heart and Vascular Centre, Hungary, Budapest. Data collection was carried out by filling in a paper–pencil questionnaire during patients’ regular control cardiology visits. Before receiving the questionnaire, one of the team members informed the patients about the participation conditions and asked for informed written consent. We did not use any further incentives. The inclusion criteria for selecting participants were as follows: at least three months had passed since the transplant surgery, and the participant provided informed consent. The exclusion criteria for filling out the anonymized questionnaires were either cognitive or physical disabilities.

### 2.2. Measurement

We assessed demographic variables such as age, sex, social variables such as relationship status, number of years spent in education, and illness-related variables, such as the time since heart transplantation.

We considered alcohol consumption and depression as psychological variables. We measured self-reported alcohol use by the Alcohol Use Disorder Identification Test (AUDIT), where a higher score indicates more problematic alcohol consumption [15]. The AUDIT showed acceptable internal consistency (Cronbach’s alpha = 0.732) in our sample. We used four categories based on the AUDIT scores: abstinence (AUDIT = 0), low-risk drinking (AUDIT score 1–7), a medium level of alcohol problems (AUDIT score 8–15), and a high level of alcohol problems (AUDIT score more than 15) [15,16]. We defined at-risk drinking based on the first three questions of the AUDIT (AUDIT-C) [17]. At-risk drinking is shown by an AUDIT-C scoring 4 or more for men and 3 or higher for women [6]. The AUDIT and AUDIT-C questionnaires were considered scorable if all items were completed or in cases where only item 2 was left unanswered, provided that the combined score of items 1 and 3 was zero. At the same time, we used the 9-item Beck Depression Inventory (BDI*-*9) [18], where higher total scores indicate more severe depressive symptoms. The questionnaire showed good reliability (Cronbach’s alpha = 0.827) in our study.

We considered health literacy and medication adherence as health-related variables. We assessed self-reported health literacy using the Brief Health Literacy 4-question screening tool (BRIEF), where a higher score indicates a higher level of health literacy [19]. In our sample, all four items had low reliability (Cronbach’s alpha = 0.562); therefore, based on the further item-total statistics, we decided to use only the first three questions, deleting the item “How confident do you feel when you fill out forms about your health status independently?” (Cronbach’s alpha = 0.795).

We measured the medication adherence using a modified version of the Medication Adherence Report Scale (MARS-5) [20] for immunosuppressive medication adherence, in which a higher score indicates a higher level of adherence. The questionnaire showed good reliability (Cronbach’s alpha = 0.814) in our sample.

The database was analyzed using IBM SPSS 29 statistical software, applying descriptive statistics and Pearson’s correlation test. Multivariate regression analysis was conducted using the Enter method. The internal reliability of the psychological questionnaires was assessed using Cronbach’s alpha coefficient. During the preparation of this manuscript, the authors used language editing AI tool, the authors have reviewed and edited the output and take full responsibility for the content of this publication.

## 3. Results

We enrolled 201 heart transplant recipients; the baseline characteristics of the sample are shown in Table 1.

### 3.1. Scorability of the Questionnaires

**Figure 1:**
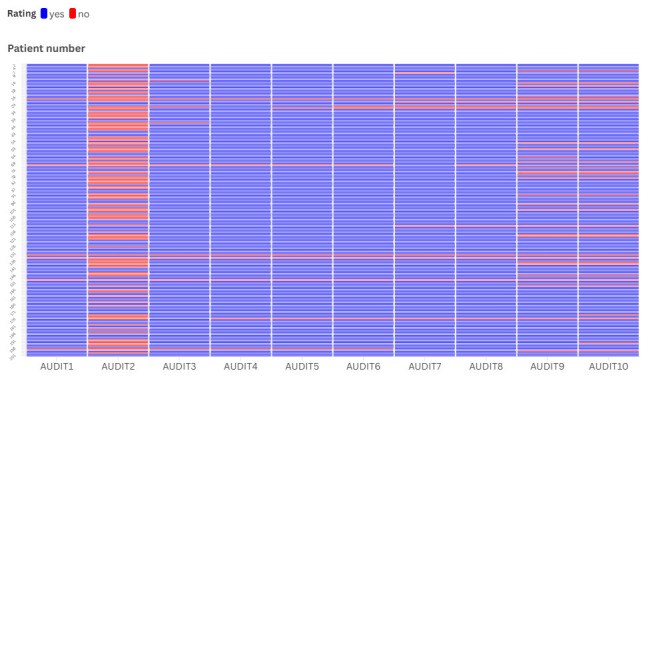
Heatmap of individual answers to the AUDIT questions.

The AUDIT questionnaire was not assessable for 19.9% (*n* = 40) of the respondents. Within this category, 1.9% (*n* = 4) skipped the entire questionnaire. The AUDIT-C score computation was not feasible in 6.0% (*n* = 12) of the sample. However, the nonscorability of the BRIEF questionnaire was only 5.5% (*n* = 11), that of the MARS-5 questionnaire modified for immunosuppression was 9% (*n* = 18), and that of the BDI*-*9 questionnaire was 6.4% (*n* = 13) in our sample. Due to the relatively high rate of nonscorability, we visualized the individual answers to each AUDIT question for deeper understanding (Figure 1).

### 3.2. Descriptive Results

Out of a maximum possible score of 40 points, the mean AUDIT score in our assessable sample was 1.65 ± 2.53 (minimum 0, maximum 15). A total of 41.0% (*n* = 66) of patients were classified as abstinent (AUDIT = 0), while 54.7% (*n* = 88) were classified as low-risk consumers (AUDIT score 1–7), and 4.3% (*n* = 7) had a medium level of alcohol problems (AUDIT score 8–15). None of our patients had high level of alcohol problems (AUDIT score above 15) (Table 2).

The average AUDIT-C score was 1.03 ± 1.46 (minimum 0, maximum 8) in our scorable sample. We found that 6.5% (*n* = 13) of the respondents were at-risk drinkers (Table 3). The distribution of responses to the individual AUDIT-C questionnaire items is presented in Appendix A. We found that 11.9% (*n* = 23) of respondents reported consuming six or more alcoholic drinks on a single occasion either monthly or less than monthly but not never.

Out of a maximum of 20 points, the respondents received an average BRIEF score of 14.7 ± 0.2 (min. 5, max. 20). Among the respondents, 14.9% (*n* = 30) had inadequate, 59.7% (*n* = 120) had problematic, and 19.9% (*n* = 40) had adequate health literacy. The average modified MARS-5 score was 24.6 ± 1.2 (min. 14, max. 25). The average depression score was 2.9 ± 3.3 (min. 0, max. 19). Based on these results, 88.6% (*n* = 178) of the sample exhibited a normal mood state, 4.5% (*n* = 9) showed signs of mild depression, and 0.5% (*n* = 1) showed indications of moderate depression.

### 3.3. Correlations

**Figure 2:**
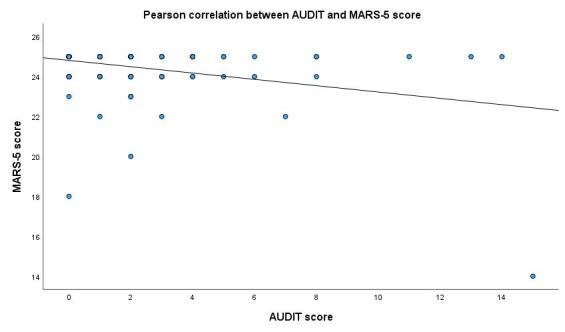
Correlation between AUDIT score and MARS-5 modified to immunosuppression score. AUDIT: Alcohol Use Disorders Identification Test, MARS-5: Medication Adherence Report Scale modified to immunosuppression. y = 24.81 − 0.16 × x.

**Figure 3:**
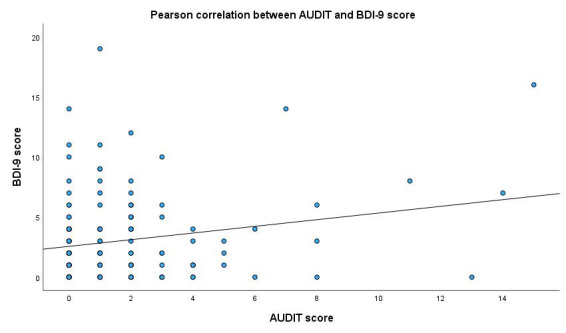
Correlation between AUDIT score and BDI-9 score. AUDIT: Alcohol Use Disorders Identification Test, BDI-9: 9-item Beck Depression Inventory. y = 2.56 + 0.28 × x.

**Figure 4:**
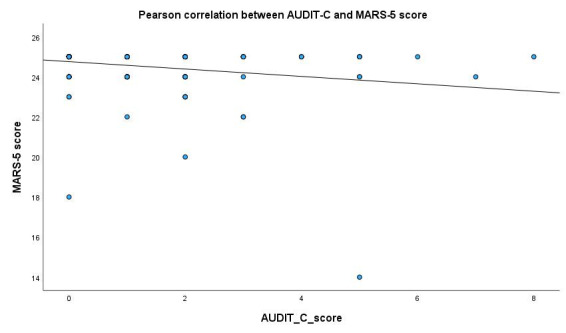
Correlation between AUDIT-C score and MARS-5 modified to immunosuppression score. AUDIT-C: Alcohol Use Disorders Identification Test−Consumption, MARS-5: Medication Adherence Report Scale modified to immunosuppression. y = 24.76 − 0.18 × x.

The scorability of the AUDIT was independent of age, sex, time since HTX, education, marital status, and BDI-9, BRIEF, and modified MARS-5 scores. The AUDIT score showed a weak but significant negative correlation with medication adherence to immunosuppressive therapy (r = −0.326; *p* < 0.001; *n* = 147) (Figure 2) and a significant positive correlation with depression scores (r = 0.208; *p* = 0.010; *n* = 154) (Figure 3). The AUDIT score also exhibited a very weak negligible negative correlation with health literacy (r = −0.176; *p* = 0.027; *n* = 157). We found no significant correlation with age, education, or time since HTX. However, there was a significant difference in AUDIT scores based on sex (t (121, 140) = −3.127; *p* = 0.002), with male respondents receiving higher scores. We found a significant negative correlation between the AUDIT-C score and medication adherence to immunosuppressive therapy (r = −0.231; *p* = 0.002; *n* = 147) (Figure 4). There was no significant correlation between AUDIT-C and BDI-9 scores (r = 0.108; *p* = 0.149; *n* = 180).

### 3.4. Multivariate Regression Analysis

Multivariate regression analyses revealed that our models (see Table 4 and Table 5) significantly predicted both the AUDIT score (F = 5.106; *p* < 0.001), explaining 21.6% of its variance (R^2^ = 0.216), and the AUDIT-C score (F = 3.804; *p* = 0.002), explaining 14.6% of the variance (R^2^ = 0.146). Sex and medication adherence were significant independent predictors in both models.

## 4. Discussion

To our knowledge, there are no available national data concerning alcohol consumption patterns among heart transplant recipients or other solid organ transplant populations, and international data are also limited. In our sample, the prevalence of alcohol consumption and at-risk consumers was consistently lower than the previously published data: the prevalence of alcohol consumption in our sample was 59%, with 6.5% at-risk drinkers, whereas Verhalle et al. found an 80.8% consumer and 27.6% at-risk drinker level among 130 heart transplant recipients [6]. Similarly, 15% at-risk drinkers was reported in the broader solid organ transplant population [13]; furthermore, a meta-analysis by Dobbels et al. identified heart transplant recipients as exhibiting the highest rate of alcohol consumption among solid organ transplant recipients, with an annual incidence of 0.045 cases [14]. This discrepancy may be caused by the fact that 19.9% of patients did not complete the AUDIT questionnaire in a scorable manner, a notably higher rate than that observed for the other instruments in the assessment battery. This finding suggests a relatively high rate of noncompletion that is specific to the AUDIT. Despite our anonymization, the sensitivity of the topic may contribute to this, as may methodological factors. Although most studies use the single-administered AUDIT [21], its incorporation into longer health surveys or into our multi-instrument format appears to increase the frequency of nonscorable responses. Supporting this, Daeppen et al. reported that 33% of their recruited participants declined to complete the AUDIT when it was incorporated into a longer health questionnaire (H-AUDIT); however, the same participants did complete the single-administered AUDIT [22]. Our finding can be clinically relevant, because the current guidelines recommend pretransplant screening with AUDIT, as well as post-transplant regular follow-up [7].

In our population, 11.9% of the respondents had consumed six or more drinks at least once. This consumption pattern may affect the transplanted graft in multiple ways. In general, heavy episodic or binge drinking can cause myocardial injury, where even just a single binge-drinking episode may elicit an inflammatory response in the myocardium [23,24]. On the other hand, this kind of consumption potentially impairs the liver function [25], which, in turn, can affect the metabolism of antirejection drugs such as tacrolimus by decreasing the clearance and altering elimination [26]. Due to its clinical relevance, we suggest the screening for binge drinking pattern in this special population. AUDIT items 2 and 3 provide more detailed information about this pattern, beyond the overall score.

The relationship between alcohol use and post-transplant outcomes is most extensively studied in the liver transplant population [12]. We observed weak but significant correlations between the AUDIT and AUDIT-C scores and adherence to immunosuppressive therapy, which is a critical message for transplant specialists. Prior research indicated that recipients with a history of substance abuse showed a significantly higher prevalence of noncompliance and associated mortality over a long-term follow-up [13]. Our results—such as at-risk drinking’s correlation with adherence to immunosuppressive therapy—imply that even subclinical alcohol use may compromise medication adherence, warranting further attention for clinicians. In a systematic review of chronic disease patient populations, most studies reported negative effects of alcohol consumption on adherence [21]. The authors highlight the need for further research using validated measurements, which assess different alcohol consumption patterns and objectively measured adherence. Nevertheless, the clinical relevance of at-risk drinking is still not clear in heart transplant population.

We also observed a significant correlation between depression and the AUDIT score, in accordance with the existing international evidence [27,28,29]. Given that depression is nearly twice as prevalent among heart transplant recipients as in the general population, our results highlight the critical need for comprehensive psychological assessment, including routine screening for depressive symptoms, as part of the standard post-transplant follow-up protocol.

Our results point out a more complex psychosocial follow-up in the heart transplant population including a more nuanced assessment of alcohol consumption patterns. Our further findings support the WHO point of view about “no safe alcohol consumption”, rather than the current ISHLT guideline of heart transplant care [30], which recommends limiting alcohol intake to 1–2 drinks per day and cites the national recommendations regarding “safe drinking”. As an intervention for non-safe drinkers, it suggests consultation on the harmful effects of alcohol. In our institution, we use the concept of psychologically informed care [31], in which the multidisciplinary team provides psychosocial support by a health psychologist, clinical psychologist, addiction counselor, and a psychiatrist, and the medial doctors are trained in low-intensity psychological interventions as well. Our approach aligns with the results of Sollenberger et al.’s meta-analysis, who found that psychosocial therapy for AUD patients provided by a collaborative multidisciplinary team was more pronounced than that by a single provider independently [32].

Further research is needed in the heart transplant population to uncover alcohol consumption patterns and its associations with post-transplant clinical outcomes in order to determine evidence-based recommendations for effective interventions.

## 5. Limitations

As with any cross-sectional design, this study has inherent limitations that warrant consideration. Specifically, the cross-sectional nature precludes the ability to capture changes over time in key variables such as alcohol consumption and depressive symptomatology. As a result, potential longitudinal relationships between these factors and important clinical outcomes remain unexplored. Our data collection was carried out by only one center, with a relatively high proportion of nonscorable AUDIT questionnaire, which also limits the generalizability of the results. We would also note the lack of potential confounders, such as clinical data, history of substance use disorder, and pharmacotherapy differences. Our conclusions may apply only with the consideration of these limitations.

## 6. Conclusions

The observed association between alcohol consumption patterns and adherence to immunosuppressive therapy emphasizes the importance of targeted screening in the long-term care of heart transplant recipients.

Although the AUDIT remains a valuable screening tool, its effectiveness may be compromised when administered as part of a larger multi-instrument survey. Our findings suggest that the standalone administration of the AUDIT may yield higher response rates and greater clinical utility. Furthermore, beyond the total AUDIT score, clinicians should pay close attention to both at-risk drinking and high-risk consumption patterns, such as binge drinking, particularly in patients receiving liver-metabolized immunosuppressants.

Although the prevalence of at-risk drinking in our cohort was relatively low, its potential impact on transplant outcomes warrants further investigation. Our study supports the integration of specialized mental health service providers—including addiction specialists—into the multidisciplinary teams managing heart transplant patients, a recommendation that is scarcely mentioned in the current literature [33]. In conclusion, comprehensive screening for alcohol use, depression, and medication adherence should be an integral part of post-transplant care to optimize the long-term outcomes in this vulnerable population.

## Figures and Tables

**Table 1:** Baseline characteristics of the sample.

Variables	
**Sex**	*n* (%)
Male	152 (75.60)
Female	49 (24.40)
**Age, years** (mean ± SD; min, max)	56.33 ± 11.46 (min. 19, max. 80)
**Time since transplant, years** (mean ± SD; min, max)	6.37 ± 4.04 (min. 0, max. 27)
**Years spent in education, years** (mean ± SD; min, max)	13.31 ± 3.33 (min. 3, max. 25)
**Relationship status**	*n* (%)
Married or in a relationship	142 (70.64)
No partner	58 (28.86)
Missing	1 (0.5)
**Health-related variables**	Mean ± SD (min, max)
BRIEF score	9.60 ± 2.24 (min. 2, max. 12)
Modified MARS-5 score	24.58 ± 1.16 (min. 14, max. 25)
BDI-9 score	2.90 ± 3.31 (min. 0, max. 19)

BRIEF: Brief Health Literacy Screening Tool, MARS-5: Medication Adherence Report Scale modified to immunosuppression, BDI-9: 9-item Beck Depression Inventory, *n*: number of participants, SD: standard deviation.

**Table 2:** Descriptive statistics of AUDIT categories.

AUDIT Score	AUDIT Category	% (*n*)	Mean ± SD	Minimum	Maximum
0	Abstinent	41.0% (*n* = 66)	-	-	-
1–7	Low-risk	54.7% (*n* = 88)	2.14 ± 1.35	1	7
8–15	Medium-risk	4.3% (*n* = 7)	11.00 ± 3.06	8	15
>15	High-risk	0% (*n* = 0)	-	-	-

AUDIT: Alcohol Use Disorders Identification Test, *n*: number of participants, SD: standard deviation.

**Table 3:** Descriptive statistics of AUDIT-C categories.

AUDIT-C Score	AUDIT-C Category	% (*n*)	Mean ± SD	Minimum	Maximum
0–2 (female) 0–3 (male)	No risk	93.1% (*n* = 176)	0.73 ± 0.94	0	3
>2 (female) >3 (male)	At-risk	6.9% (*n* = 13)	5.08 ± 1.32	3	8

AUDIT: Alcohol Use Disorders Identification Test, AUDIT-C: Alcohol Use Disorders Identification Test–Consumption, *n*: number of participants, SD: standard deviation.

**Table 4:** Linear regression analysis, dependent variable: AUDIT score.

Predictor	B	SE	β	t	*p*
Intercept	17.903	4.431	—	4.040	<0.001
Sex (1 = male, 0 = female)	1.048	0.496	0.185	2.111	0.037
In a relationship (1 = yes)	0.023	0.472	0.004	0.049	0.961
Years of education	0.035	0.071	0.044	0.491	0.624
BDI-9 score	0.050	0.072	0.071	0.691	0.491
MARS-5 score	−0.673	0.162	−0.373	−4.161	<0.001
BRIEF 3-item total	−0.114	0.118	−0.096	−0.965	0.337

BRIEF: Brief Health Literacy Screening Tool; MARS-5: Medication Adherence Report Scale modified to immunosuppression; BDI-9: 9-item Beck Depression Inventory; B: unstandardized coefficient; SE: standard error; β: Standardized coefficient; t: t-value; *p:* probability value.

**Table 5:** Linear regression analysis, dependent variable: AUDIT-C score.

Predictor	B	SE	β	t	*p*
Intercept	7.188	2.534	—	2.836	0.005
Sex (1 = male, 0 = female)	0.722	0.268	0.224	2.694	0.008
In a relationship (1 = yes)	0.106	0.253	0.035	0.418	0.676
Years of education	0.048	0.039	0.106	1.253	0.212
BDI-9 score	0.024	0.040	0.060	0.614	0.540
MARS-5 score	−0.288	0.093	−0.263	−3.104	0.002
BRIEF 3-item total	−0.042	0.061	−0.065	−0.697	0.487

BRIEF: Brief Health Literacy Screening Tool; MARS-5: Medication Adherence Report Scale modified to immunosuppression; BDI-9: 9-item Beck Depression Inventory; B: unstandardized coefficient; SE: standard error; β: Standardized coefficient; t: t-value; *p:* probability value.

**Figure A1:**
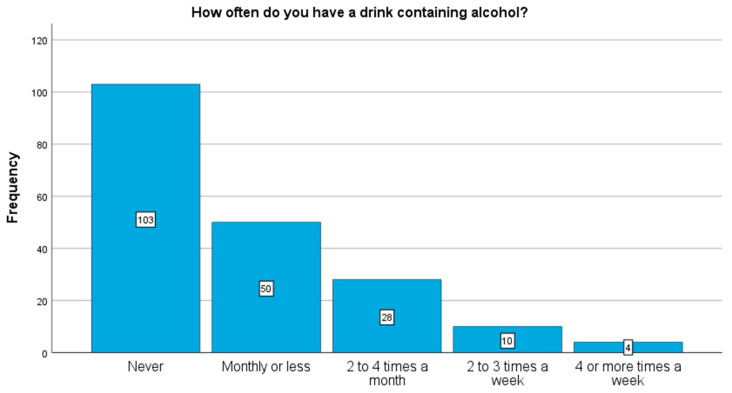
Distribution of Answers to the First Item of the AUDIT.

**Figure A2:**
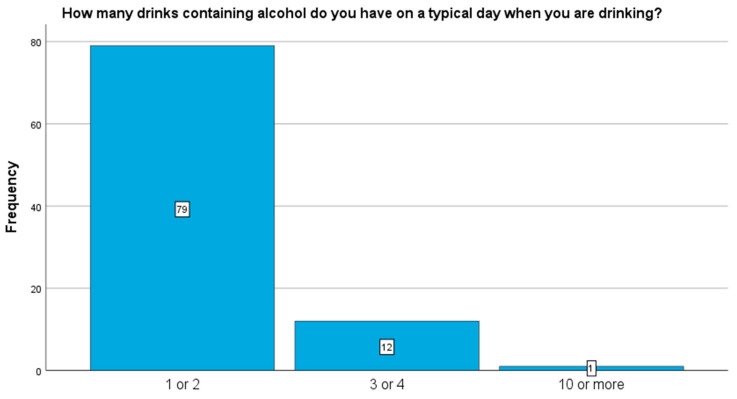
Distribution of Answers to the Second Item of the AUDIT.

**Figure A3:**
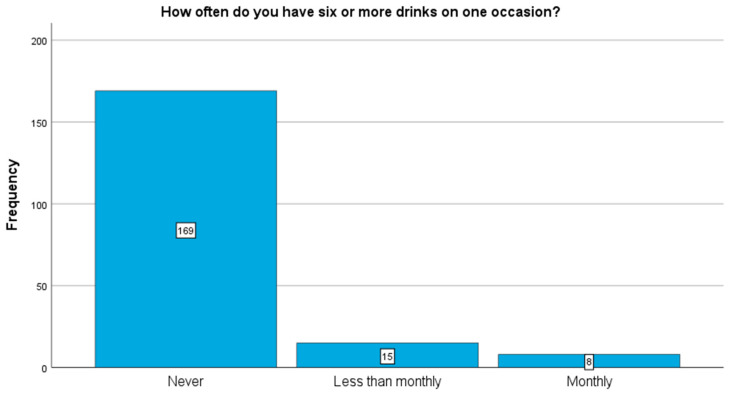
Distribution of Answers to the Third Item of the AUDIT.

## Data Availability

Dataset available on request from the authors. The data are not publicly available due to privacy reasons.

## Abbreviations

The following abbreviations are used in this manuscript:

AUDIT	Alcohol Use Disorders Identification Test
AUDIT-C	Alcohol Use Disorders Identification Test–Consumption
BRIEF	Brief Health Literacy Screening Tool
MARS-5	Medication Adherence Report Scale
BDI*-9*	9-item Beck Depression Inventory
AUD	Alcohol Use Disorder
